# Effects of single- and dual-task training on dual-task walking performance, prosthetic-limb dynamics and quality of life in unilateral transtibial amputees

**DOI:** 10.3389/fspor.2026.1944825

**Published:** 2026-09-03

**Authors:** Faezeh Saeidi Rashkoliya, Fariborz Hovanloo, Mehdi Gheitasi, Paul William Macdermid

**Affiliations:** 1 Department of Health & Sports Rehabilitation, Faculty of Sport Science & Health, Shahid Beheshti University, Tehran, Iran; 2 School of Sport, Exercise and Nutrition, College of Health, Massey University, Palmerston North, New Zealand

**Keywords:** amputation, dual-task performance, dual-task training, gait, quality of life, single-task training

## Abstract

**Introduction:**

The aim of the study was to compare the effects of single-task versus dual-task training on dual-task walking performance, prosthetic-limb kinematics, centre-of-pressure behaviour, and health-related quality of life in adults with transtibial amputation.

**Methods:**

Forty-five participants undertook an eight-week intervention and were randomly allocated to one of three groups: control, single-task training, or dual-task training. Gait variables were obtained from 3D motion capture and force-plate analysis, while health-related quality of life was assessed using a composite measure derived from the WHOQOL-BREF.

**Results:**

Two-way mixed ANOVA showed that both single-task and dual-task training produced significant improvements in dual-task walk-performance time compared with the control group (*p* < 0.0001). Positive training effects were also observed for prosthetic-limb step length (*p* < 0.001), knee and hip joint range of motion (*p* < 0.05), and anterior-posterior (*p* < 0.001) and mediolateral (*p* < 0.01) centre-of-pressure displacement, with dual-task training producing greater improvements than single-task training across all gait variables. Only dual-task training resulted in significant pre- to post-intervention improvements in participants quality of life (*p* < 0.0001).

**Discussion:**

These findings indicate that an eight-week programme of single- or dual-task training improves walking capability in individuals with transtibial amputation, reflective in improved gait automaticity and more stable, efficient centre-of-pressure transitions from foot strike to push-off in the affected limb. Dual-task training provides additional benefit, producing greater gains across all gait variables and resulting in a significant improvement in perceived quality of life. Future research should examine the long-term transfer of cognitive-motor training to real-world mobility and participation outcomes.

## 1 Introduction

Limb amputation is often an unavoidable procedure performed in advanced stages of conditions such as diabetes mellitus, peripheral arterial occlusive disease, malignant disorders, trauma, infection, or war-related injury (1–3). These conditions profoundly affect quality of life (4, 5) through reduced mobility, pain, and loss of bodily integrity (6, 7). Modern prosthetic devices provide a safe and effective means of restoring functional mobility for transtibial (below-knee) amputation, helping to mitigate some of the negative consequences of limb loss (8). The successful use of such devices depends on access to structured rehabilitation and prosthetic-specific training in the care setting (9). These interventions aim to improve postural instability (10, 11), weakness of the hip stabilizers (12), and thigh muscle atrophy (13). During walking they demonstrate increased energy expenditure, reduced walking speed, and marked gait asymmetry with diminished sensory feedback (14–17). These impairments contribute to restricted mobility (18) and a high incidence of falls (19–21), despite the use of prosthetic devices.

As such, functional limitations arise from several underlying sensorimotor deficits. The loss of ankle-foot musculature (22–24) reduces distal proprioceptive input (25, 26), requiring individuals to actively control foot placement and weight shifting (27, 28). As a result, propulsive forces are generated primarily by the proximal musculature of the hip and thigh rather than the ankle (13, 29), even though modern prosthetic feet can contribute some forward propulsion when appropriately loaded (30). Together, these sensorimotor deficits increase the attentional demands of walking, as individuals must consciously monitor limb position and stability thus reducing gait automaticity (31, 32). The elevated reliance on cognitive resources reduces the capacity available for simultaneous tasks and has been associated with reduced gait automaticity, fear of falling and fall events (33, 34).

Reducing the relative cognitive demand by increasing cognitive capability or greater automaticity of movement (35) may help improve quality of life and enhance safety in this clinical population. Consequently, rehabilitation methods that enhance the ability to allocate attentional resources and coordinate multiple tasks simultaneously (36–38), referred to as dual-task training, could be beneficial for everyday tasks such as walking while maintaining one's balance (39). Dual-task methods are also used to assess the concurrent performance of a postural-task combined with a cognitive task, providing insight into how cognitive processes, such as attention and executive function, interact with postural control while standing and walking (40).

Previous work in lower-limb amputees has shown that single-task training primarily enhances single-task performance, whereas dual-task-based rehabilitation can improve dual-task postural control in people with transfemoral amputations (35). These findings suggest that incorporating cognitive-motor demands into rehabilitation may have relevance for everyday functional activities. Therefore, the aim of this study was to compare the effects of single-task versus dual-task training on dual-task walking performance, prosthetic-limb kinematics, centre-of-pressure behaviour, and health-related quality of life in community-ambulating (K3-K4) adults with transtibial amputation. It was hypothesized that dual-task training would elicit greater improvements across all outcomes, reflecting the added demands on cognitive-motor integration and gait automaticity.

## 2 Materials and methods

### 2.1 Participants

Fifty-six adults with unilateral transtibial amputation screened for eligibility. Four did not meet the inclusion criteria, and two declined participation, leaving fifty eligible participants. These participants had a body mass index between 22 and 29, were at least five years post-amputation, continuous prosthesis use for at least one year, a Montreal Cognitive Assessment score >21, no systemic medical conditions affecting gait, the ability to walk at least 10 m without assistance, and no neurological, cognitive, or orthopaedic disorders, or history of total hip replacement. Participants used their own prescribed prostheses during testing and training, and none reported changes to prosthetic components in the 12-month period prior to data collection, ensuring stable acclimation to their prosthesis. All prostheses used supracondylar suspension systems and solid-ankle cushioned heel (SACH) feet, a non-articulated foot design typically prescribed for low-activity users. All participants provided written informed consent in accordance with the University Human Ethics Committee and principles of the Declaration of Helsinki. This sample size was based on priori statistics (G*Power 3.1.9.2, Heinrich-Heine University, Dusseldorf, Germany) for analysis of variance (ANOVA) with a within-between interaction. Parameters were set at *α* = 0.05, *β* = 0.10 (powe*r* = 0.90), and an effect size of d = 0.6 based on pilot data from the walking speed test. This analysis indicated that at least 11 participants per group were required. To account for potential dropouts, 15 participants were recruited per group.

### 2.2 Procedures and measurements

Before the study began and to remove selection bias, participants attended an orientation session during which the study procedures were fully explained. Participants were then randomly assigned to one of three groups using simple randomisation in IBM SPSS Statistics (Version 22.0, IBM Corp., Armonk, NY). A computer-generated random variable was created, participants were sorted according to that variable, and then assigned sequentially to the single-task group (*n* = 15; age 42.7 ± 4 years; Weight 82.1 ± 6.0 kg; height 174 ± 6 cm; BMI 26.9 ± 1.0), dual-task group (*n* = 15; age 41.6 ± 6.9 years; Weight 83.1 ± 7.2 kg; height 174 ± 7 cm; BMI 27.5 ± 1.3), and control group (*n* = 15; age 41.4 ± 5.8 years; Weight 82.5 ± 8.0 kg; height 175 ± 8 cm; BMI 26.9 ± 1.9). All participants completed pre-test (initial) assessments including the walk performance test with gait assessment, and the Quality-of-Life assessment prior to the intervention.

**Figure 1:**
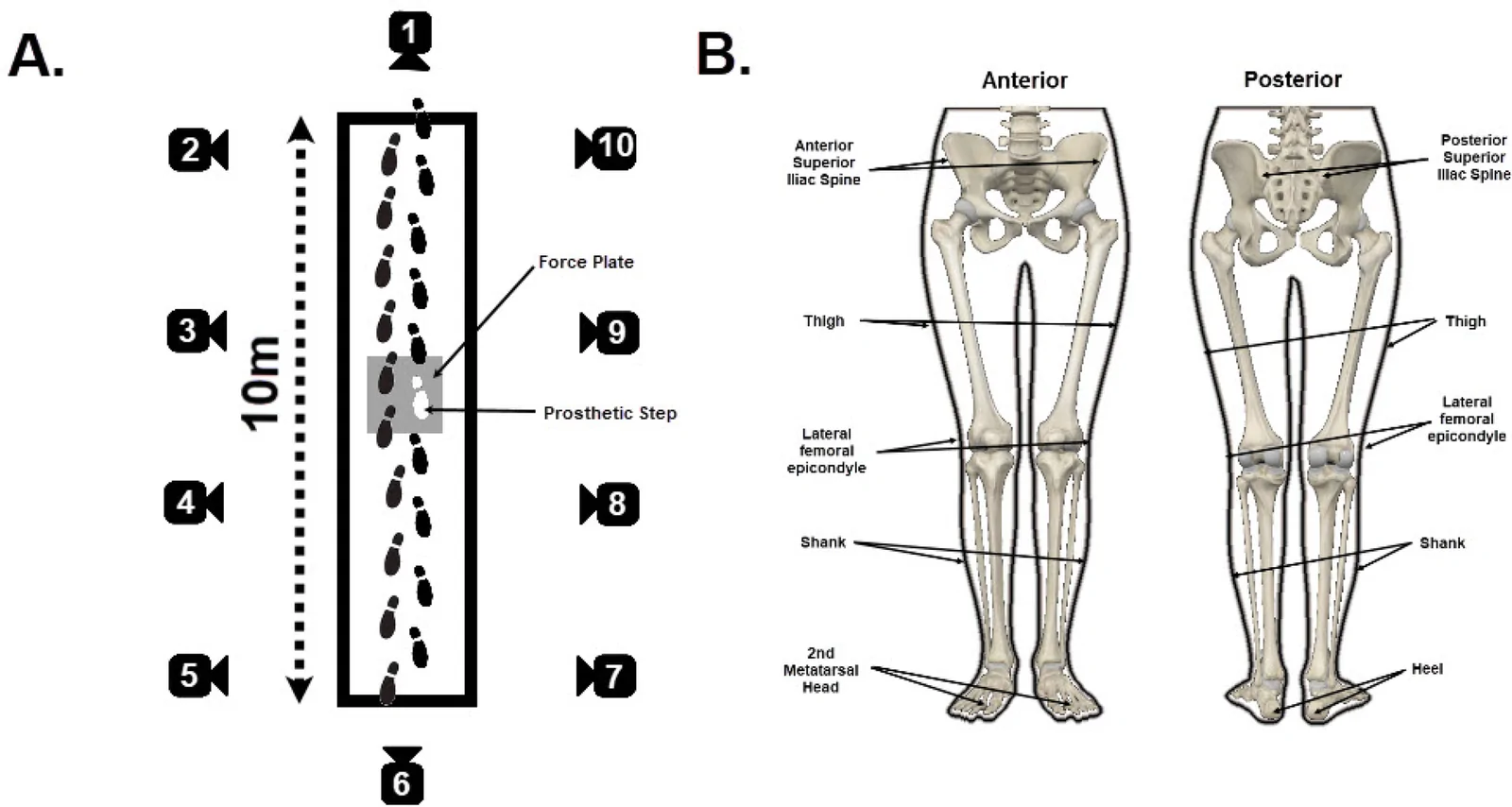
**(A)** Schematic layout of the walk track. **(B)** Marker placement.

The timed walk test (Figure 1A) involved participants walking a 10 m distance at their normal self-selected speed over a 1,000 Hz force plate (Kistler Instrumente AG, Winterthur, CH) placed at 5 m into the walk. A single force plate was used sole to obtain centre-of-pressure data for the prosthetic limb. Participants were recorded using a 10-camera motion-capture system (Vicon Motion Systems Ltd, Oxford, UK) operating at 240 Hz and placed at either end of the walkway and at 3 m intervals in its length. During each trial, participants performed an additional cognitive task that required counting backwards in threes from a randomly selected number between 100 and 200 (41, 42). Prior to data collection, participants completed several familiarization walking trials to reduce learning effect and to adjust to the testing environment. Each participant then completed three valid walking trials where the prosthetic limb contacted the force plate. For this test, reflective markers were positioned on key anatomical landmarks by the same trained researcher following standardised palpation procedures specified in the Plug-in Gait Model, developed by Vicon Motion Systems Ltd (43). The motion-capture system underwent full calibration at the start of each testing session, including static and dynamic IR wand calibration for volume, and camera alignment. Marker placement included the second metatarsal head, lateral malleolus, heel, shank, lateral femoral condyle, thigh, and both the anterior superior and posterior superior iliac spines (Figure 1B). These placements were visually verified, and a static participant calibration trial was recorded within the calibrated volume prior to each walking trial to ensure no displacement had occurred.

Participants' walk performance time (s) to complete each trial were recorded and the average of the three trials was reported to limit variability. Data from the motion capture software and force plate were processed using MATLAB (R2022b, MathWorks Inc., Natick, MA, USA) where a low-pass Butterworth filter with a cutoff frequency of 10 Hz was applied. Prosthetic step length (m) was calculated from the motion-capture data using the Plug-in Gait gait-event detection. Step length was defined as the longitudinal distance from heel strike of sound limb to the heel strike of the prosthetic limb and was converted to a non-dimension normalised value based on step length (m) divided by the prosthetic limb length (residual limb plus prosthesis) (44). Based on the Plug-in Gait marker system (Figure 1B), joint angles were computed using procedures previously described (45). The joint range of motion was obtained by subtracting the minimum recorded joint angle from the maximum angle during each gait cycle. Ground reaction force data from the embedded force plate were converted into anterior-posterior and mediolateral centre-of-pressure displacements (mm).

Health related quality-of-life was assessed using the WHOQOL-BREF, a 26-item questionnaire covering physical health, psychological health, social relationships, and environmental factors. In this study, a composite score was calculated by summing all 26-items, providing a total score ranging from 26 to 130, with higher scores indicating a better quality of life.

### 2.3 Intervention

The intervention for the single- and dual-task groups consisted of 8-weeks of training (Table 1), delivered three times per week, with each session lasting 60 min. The single-task group completed motor training focused on body stability and mobility exercises (35). From the third week onward, balance exercises and outdoor walking were added to increase functional performance on uneven surfaces (35). The dual-task group performed the same motor exercises but with concurrent cognitive tasks. The first session was performed without cognitive load to ensure task familiarity, after which cognitive activities of increasing difficulty were introduced, including backward counting, arithmetic, reverse-order sentence repetition, and verbal fluency tasks (35, 46). Participants were instructed to allocate equal attention to both motor and cognitive components. The control group did not receive any structured training and were instructed to maintain their usual daily activities throughout the study period.

**Table 1:** Eight-week progressive single-task and dual-task training programme.

Weeks	Static & dynamic stability	Strength & mobility	Dual task Cognitive load
1–2	Static weight-bearing (3–5 × 10–15 s); Repeated stepping (2 × 10 steps); Walking sideways (2 × 10 m)	Pelvic tilts (3 × 15 s); Roll-downs (2 × 5 reps); Hip abductor strengthening (2 × 10 reps)	Session 2 onwards: Backward counting; simple arithmetic.
3–4	Static weight-bearing (3–5 × 15–20 s); Stepping to elevated surface, uninvolved limb (2 × 10 steps); Walking Sideways (3 × 10 m); Backward walking (3 × 15 m)	Roll-downs (3 × 5 reps); Seated leg raises (2 × 10 reps) Hip abductor strengthening (3 × 10 reps)	Reverse-order sentence repetition; simple arithmetic
5–6	Dynamic weight-shifting (3–5 × 10–15 s); Stepping to elevated surface, involved limb (2 × 10 steps); Directional changes while walking (2 × 10 m); Ramp walk-climb (3 × 10 m)	Squats (2 × 10 reps); Bridges (2 × 10 reps); Hip abductor strengthening (3 × 10 reps)	Verbal fluency (words beginning with the same letter); more challenging arithmetic
7–8	Dynamic weight-shifting (3–5 × 15–20 s); Directional changes while walking (3 × 10 m); Stair climbing (2 × 10 m) Ramp walk-climb (4 × 10 m)	Squats (3 × 10 reps); Bridges with ring (2 × 10 reps); Hip abductor strengthening (3 × 15 reps)	Verbal fluency (words ending with the same letter); more challenging arithmetic.

At the end of the intervention period, all participants completed post-test assessment using the same procedures as in the pre-test.

### 2.4 Statistical methods

Descriptive data (mean ± SD) were used to describe the variables. A two-way mixed ANOVA was employed to examine the effects of Group (single-task, dual-task, control) and Test (pre, post) on each outcome. Where significant effects were found, Sidak's *post-hoc* multiple comparison testing were performed. All analyses were conducted using GraphPad Prism v8.4 (GraphPad Software, San Diego, CA, USA).

## 3 Results

**Figure 2:**
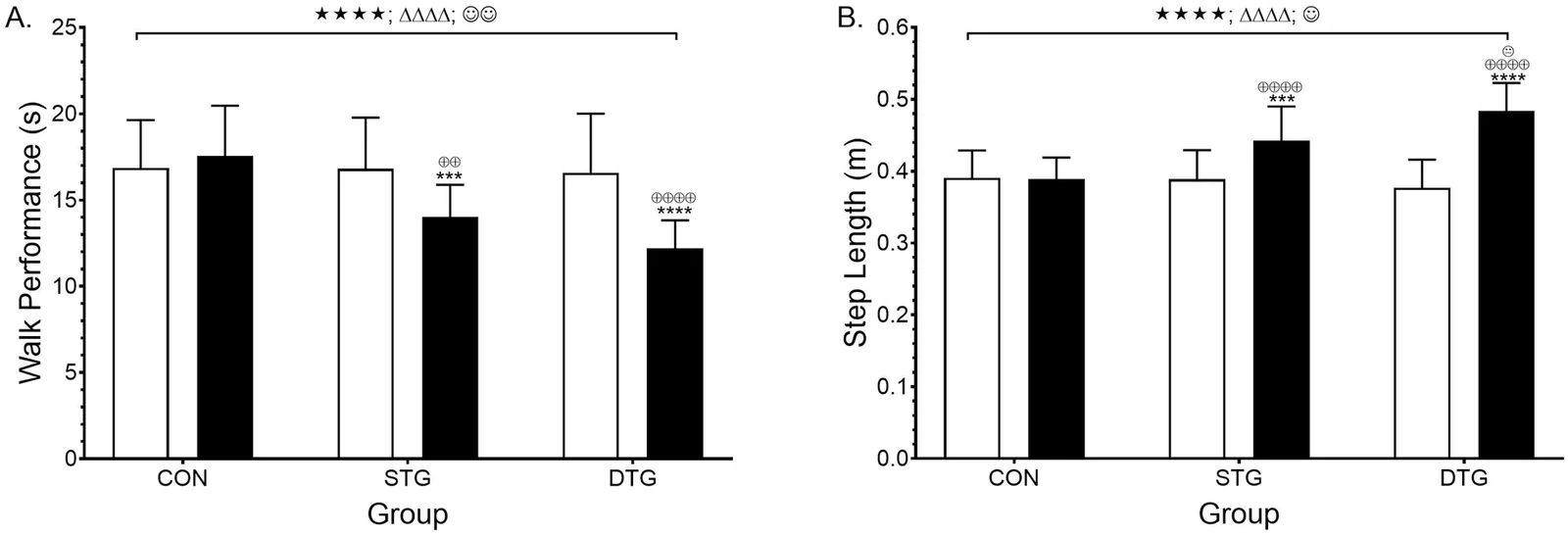
Group mean ± SD for pre- and post-test for **(A)**. Dual-task walking performance time (s) and **(B)**. Prosthesis step length (m). Where ★ signifies a group×test interaction, *Δ* is main significant main effect for test, ☺; is main effect for group, 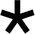 is *post-hoc* simple-effects difference between pre- and post-test for the within group, ⊕ is *post-hoc* simple-effects comparison with control and 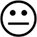 is a *post-hoc* simple-effects comparison with single-task training group.

Overall dual-task walking performance time showed a significant Group × Test interaction (F_(2,42)_ = 18.2, *p* < 0.0001, Figure 2A), along with a main effect of Test (F_(1,42)_ = 37.6, *p* < 0.0001) and Group (F_(2,42)_ = 5.32, *p* = 0.009). *Post-hoc* simple effects analyses showed no significant difference between groups for pre-test performance (*P* > 0.988). However, significant improvements from pre- to post-test were observed in the single-task group (16.81 ± 2.97 vs. 14.03 ± 1.86, *p* = 0.0001) and the dual-task group (16.58 ± 3.43 vs. 12.20 ± 1.63, *p* < 0.0001), whereas the control group showed no change (16.86 ± 2.77 vs. 17.56 ± 2.91, *p* = 0.591). Consequently, at post-test both single task and dual task groups performed better than the control group (*p* = 0.002 and *p* < 0.0001, respectively).

Prosthesis step length demonstrated a significant Group × Test interaction (F_(2,42)_ = 41.2, *p* < 0.0001, Figure 2B) with main effects of Test (F_(1,42)_ = 116.7, *p* < 0.0001) and Group (F_(2,42)_ = 4.986, *p* = 0.011). *Post-hoc* simple-effects analyses showed significant increases in prosthesis step length from pre- to post-test for the single-task group (0.39 ± 0.04 vs. 0.44 ± 0.05 m, *p* < 0.0001) and the dual-task group (0.38 ± 0.04 vs. 0.48 ± 0.04 m, *p* < 0.0001), but not the control group (0.39 ± 0.04 vs. 0.39 ± 0.03 m, *p* = 0.994). Although groups did not differ at the pre-test (*p* > 0.703), post-test prosthesis step length was greater in both the single-task group (0.44 ± 0.05 vs. 0.39 ± 0.03 m, *p* = 0.0009) and dual-task group (0.48 ± 0.04 vs. 0.39 ± 0.03 m, *p* < 0.0001) compared with the control group. In addition, post- test step length was greater in the dual-task group than in the single-task group (0.48 ± 0.04 vs. 0.44 ± 0.05 m, *p* = 0.015).

**Figure 3:**
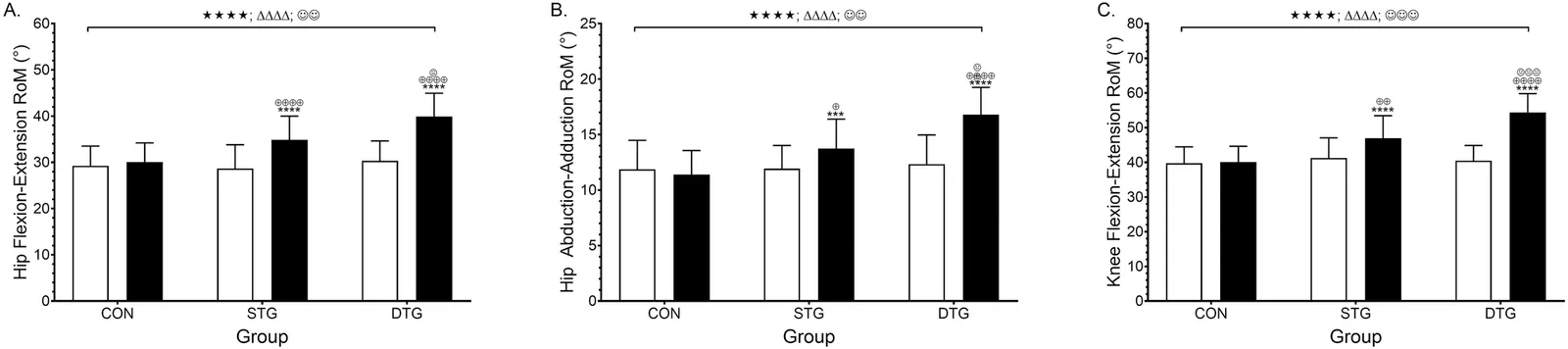
Group mean ± SD for pre- and post-test for range of motion for **(A)**. Hip Sagittal Plane (flexion-extension), **(B)**. Hip frontal plane (abduction-adduction), and **(C)**. Knee sagittal plane (flexion-extension). Where ★ signifies a group × test interaction, *Δ* is main significant main effect for test, ☺ is main effect for group, 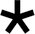 is *post-hoc* simple-effects difference between pre- and post-test for the within group, ⊕ is *post-hoc* simple-effects comparison with control and 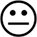 is a *post-hoc* simple-effects comparison with single-task training group.

The range of motion at the hip in the sagittal plane (Flexion-Extension) during the prosthesis step showed a significant Group × Test interaction (F_(2,42)_ = 19.37, *p* < 0.0001, Figure 3A), along with main effects of Test (F_(1,42)_ = 90.36, *p* < 0.0001) and Group (F_(2,42)_ = 6.3, *p* = 0.004). *Post-hoc* simple-effects analyses showed significant increases in the prosthesis limb range of motion in relation to flexion and extension from pre- to post-test in the single-task group (28.7 ± 5.1 vs. 34.9 ± 5.1°, *p* < 0.0001) and the dual-task group (30.3 ± 4.3 vs. 39.9 ± 5.0°, *p* < 0.0001), with no change in the control group (29.3 ± 4.2 vs. 30.1 ± 4.2°, *p* = 0.817). Groups did not differ at pre-test (*p* > 0.703), whereas post-test hip flexion-extension range of motion were greater in both the single-task group (34.9 ± 5.1 vs. 30.1 ± 4.2°, *p* = 0.019) and dual-task group (39.9 ± 5.0 vs. 30.1 ± 4.2°, *p* < 0.0001) compared with the control group. In addition, post- test hip flexion-extension ROM was less in the dual-task group than in the single-task group (39.9 ± 5.0 vs. 34.9 ± 5.1°, *p* = 0.012).

The range of motion at the hip in the frontal plane (Abduction-Adduction) during the prosthesis step showed a significant Group × Test interaction (F_(2,42)_ = 36.29, *p* < 0.0001, Figure 3B), along with main effects of Test (F_(1,42)_ = 66.7, *p* < 0.0001) and Group (F_(2,42)_ = 6.1, *p* = 0.005). *Post-hoc* simple-effects analyses showed significant increases in the prosthesis limb range of motion in relation to abduction and adduction from pre- to post-test in the single-task group (11.9 ± 2.1 vs. 13.7 ± 2.7°, *p* = 0.0002) and the dual-task group (12.3 ± 2.6 vs. 16.8 ± 2.5°, *p* < 0.0001), with no change in the control group (11.9 ± 2.6 vs. 11.4 ± 2.2°, *p* = 0.597). Groups did not differ at pre-test (*p* > 0.937), whereas post-test hip abduction-adduction range of motion were greater in both the single-task group (13.7 ± 2.7 vs. 11.4 ± 2.2°, *p* = 0.032) and dual-task group (16.8 ± 2.5 vs. 11.4 ± 2.2°, *p* < 0.0001) compared with the control group. In addition, post- test hip abduction-adduction range of motion was less in the dual-task group than in the single-task group (16.8 ± 2.5 vs. 13.7 ± 2.7°, *p* = 0.003).

The range of motion at the knee during the prosthesis step showed a significant Group × Test interaction (F_(2,42)_ = 67.91, *p* < 0.0001, Figure 3C), along with main effects of Test (F_(1,42)_ = 191.6, *p* < 0.0001) and Group (F_(2,42)_ = 8.42, *p* = 0.0008). *Post-hoc* simple-effects analyses showed significant increases in the prosthesis limb range of motion in relation to flexion and extension from pre- to post-test in the single-task group (41.3 ± 5.8 vs. 46.9 ± 6.5°, *p* < 0.0001) and the dual-task group (40.5 ± 4.4 vs. 54.4 ± 5.4°, *p* < 0.0001), with no change in the control group (39.7 ± 4.7 vs. 40.1 ± 4.6°, *p* = 0.970). Groups did not differ at pre-test (*p* > 0.937), whereas post-test knee flexion-extension range of motion was greater in both the single-task group (46.9 ± 6.5 vs. 40.1 ± 4.6°, *p* = 0.002) and dual-task group (54.4 ± 5.4 vs. 40.1 ± 4.6°, *p* < 0.0001) compared with the control group. In addition, post- test knee flexion-extension range of motion was less in the dual-task group than in the single-task group (54.4 ± 5.4 vs. 46.9 ± 6.5°, *p* = 0.007).

**Figure 4:**
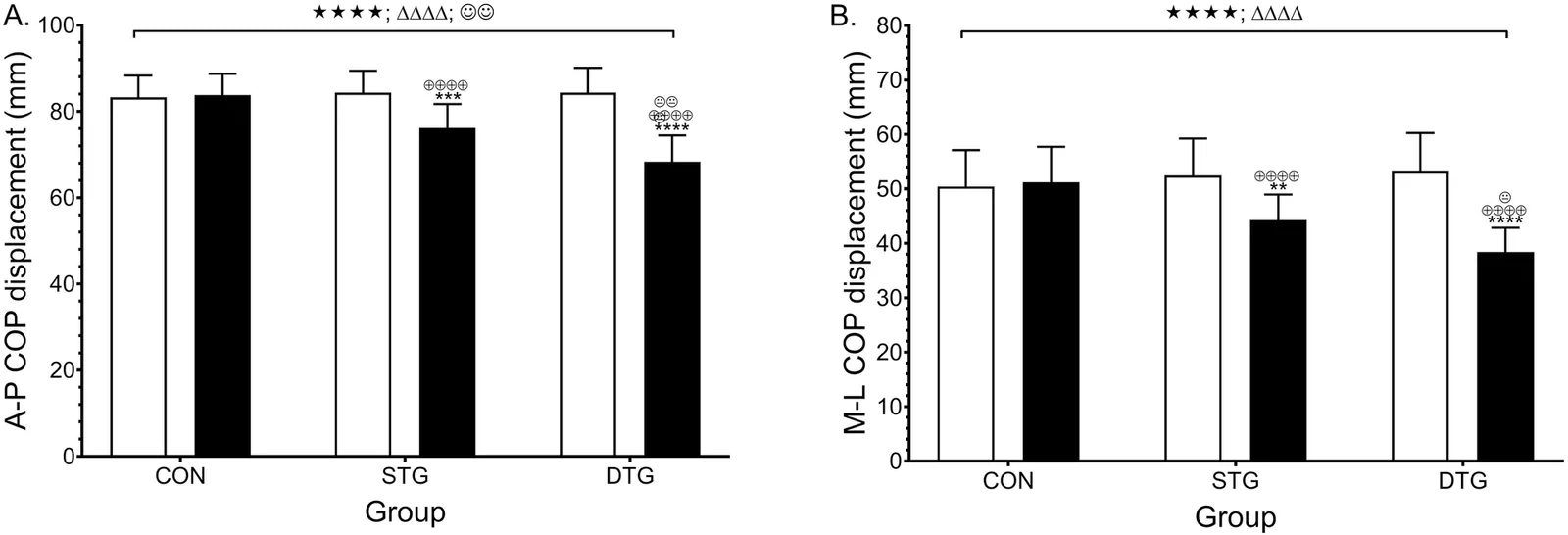
Group mean ± SD for pre- and post-test for **(A)**. Anterior-Posterior centre-of-pressure displacement (mm) and **(B)**. Medial-Lateral centre-of-pressure displacement (mm). Where ★ signifies a group × test interaction, *Δ* is main significant main effect for test, ☺ is main effect for group, 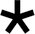 is *post-hoc* simple-effects difference between pre- and post-test for the within group, ⊕ is *post-hoc* simple-effects comparison with control and 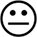 is a *post-hoc* simple-effects comparison with single-task training group.

Centre-of-pressure displacement for the prosthesis step in the anterior–posterior direction revealed a significant Group × Test interaction (F_(2,42)_ = 102.2, *p* < 0.0001, Figure 4A), along with main effects of Test (F_(1,42)_ = 277.5, *p* < 0.0001), with no main effect for Group (F_(2,42)_ = 2.9, *p* = 0.068). *Post-hoc* simple-effects analyses showed significant decreases in prosthesis anterior-posterior centre-of-pressure path length from pre- to post-test for the single-task group (84 ± 5 vs. 76 ± 6 mm, *p* < 0.0001) and the dual-task group (85 ± 6 vs. 68 ± 6 mm, *p* < 0.0001), with no change in the control group (83 ± 5 vs. 84 ± 5 mm, *p* = 0.888). Although groups did not differ at the pre-test (*p* > 0.932), post-test path length was lower in both the single-task group (76 ± 6 vs. 84 ± 5 mm, *p* = 0.0007) and dual-task group (68 ± 6 vs. 84 ± 5 mm, *p* < 0.0001) compared with the control group.

Centre-of-pressure displacement for the prosthesis step in the medial-lateral direction showed a significant Group × Test interaction (F_(2,42)_ = 47.86, *p* < 0.0001, Figure 4B), along with main effects of Test (F_(1,42)_ = 128.2, *p* < 0.0001) and Group (F_(2,42)_ = 7.23, *p* = 0.002). *Post-hoc* simple-effects analyses showed significant decreases in prosthesis medial-lateral centre-of-pressure path length from pre- to post-test in the single-task group (53 ± 7 vs. 44 ± 5 mm, *p* < 0.0001) and the dual-task group (53 ± 7 vs. 38 ± 5 mm, *p* < 0.0001), with no change in the control group (50 ± 7 vs. 51 ± 7 mm, *p* = 0.862). Groups did not differ at pre-test (*p* > 0.515), whereas post-test path lengths were less in both the single-task group (44 ± 5 vs. 51 ± 7 mm, *p* = 0.008) and dual-task group (38 ± 5 vs. 51 ± 7 mm, *p* < 0.0001) compared with the control group. In addition, post- test path length was less in the dual-task group than in the single-task group (38 ± 5 vs. 44 ± 5 mm, *p* = 0.0005).

**Figure 5:**
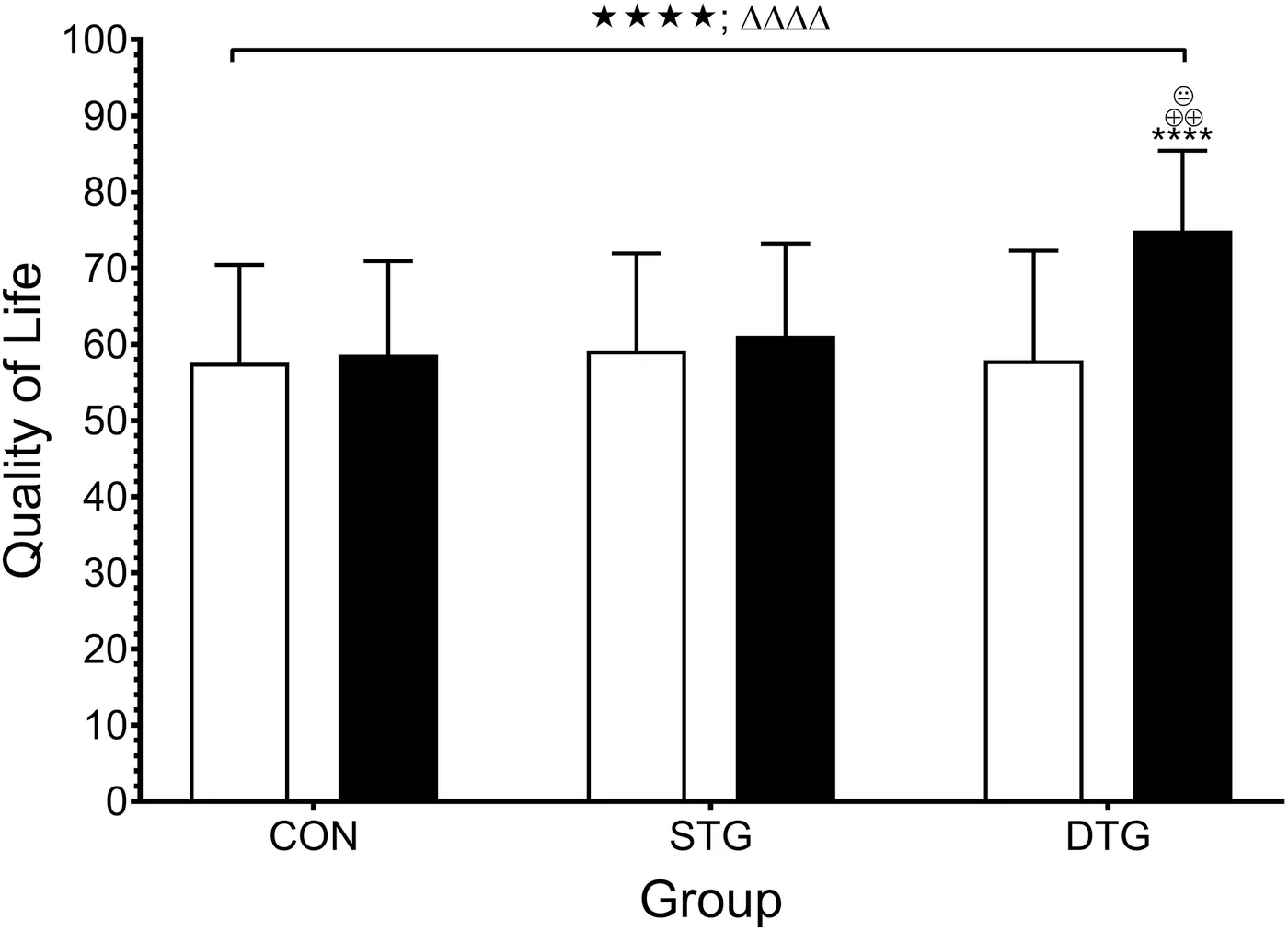
Composite quality-of-life score for each group pre- and post-intervention. Where ★ signifies a group × test interaction, *Δ* is main significant main effect for test, ☺ is main effect for group, 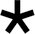 is *post-hoc* simple-effects difference between pre- and post-test for the within group, ⊕ is *post-hoc* simple-effects comparison with control and 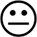 is a *post-hoc* simple-effects comparison with single-task training group.

The composite quality-of-life score showed a significant Group × Test interaction (F_(2,42)_ = 41.59, *p* < 0.0001, Figure 5) and a main effect of Test (F_(1,42)_ = 69.09, *p* < 0.0001), with no main effect of Group (F_(2,42)_ = 1.878, *p* = 0.166). Simple effects *post-hoc* analyses indicated a significant improvement from pre- to post-test in the dual-task group (58 ± 14 vs. 75 ± 11, *p* < 0.0001), with no change in the control group (58 ± 13 vs. 59 ± 12, *p* = 0.831) or the single-task group (59 ± 13 vs. 61 ± 12, *p* = 0.431).

## 4 Discussion

The aim of the study was to compare the effects of single-task and dual-task training on dual-task walking performance, prosthetic-limb kinematics, centre-of-pressure behaviour, and health-related quality of life in adults with transtibial amputation. The main findings were that following eight weeks of training, dual-task participants demonstrated the greatest overall improvements, with both groups outperforming the control group. Dual-task walking performance improved in the single- and dual-task groups accompanied by increases in prosthetic-limb step length, and greater hip and knee range of motion during walking. The prosthetic limb centre-of-pressure measures indicated reduced compensatory movement during the stance phase, characterised by reduced anterior-posterior and mediolateral path length, which is consistent movement patterns typically associated with smoother rollover and greater stance phase stability in transtibial amputees. Importantly, only the dual-task training participants quality of life scores improved.

During the intervention period, four participants withdrew due to health issues, and one participant in the control group did not attend the post-test assessment. This resulted in a total attrition of 10% for the study which is similar to that reported across 25 prosthetic intervention studies for transtibial amputation (8).

The results of this study support previous work advocating for physical therapy rehabilitation programmes in individuals with transtibial amputations (35, 47). Both single-task motor training and dual-task training were effective at improving dual-task walk performance (Figure 2A) and prosthetic-side stance-phase stability, as reflected in the reduced centre of pressure path lengths (Figure 4), despite participants having their prosthetic limb for at least one year. This convergence is unsurprising, as the motor training used (Table 1) was identical for both intervention groups and specifically targeted contributors to impaired gait in transtibial amputees. The training addressed the loss of ankle-foot musculature (19, 20, 22), reduced distal proprioception (25), and the ensuing lack of strength and postural control. However, given that transtibial amputees typically walk with elevated attentional demands due to reduced sensory feedback and increased balance requirements (10, 15, 17, 34), the balance focused motor training combined with the walk-step exercises would also be expected to reduce cognitive load and enhance gait automaticity (38). In addition to these cognitive demands, distal muscular weakness is a major contributor to reduced gait performance in individuals with transtibial amputation (13, 48), and strengthening-focused rehabilitation can effectively improve walking performance and gait mechanics when weakness is a primary limiting factor (49, 50). As such, the motor training used targeted proximal force generation through hip and thigh musculature strengthening, alongside exercises designed to enhance postural control and functional movement transitions (13, 29). Positive responses to such training have previously been associated with increased proximal muscle engagement, longer step length, and faster walking speed (49). Although this study cannot quantify muscle activation, the observed increases in step length, walking speed, and joint range of motion in both intervention groups (Figure 2) are consistent with proximal muscle engagement. Importantly, they are also expected responses to faster walking in transtibial amputees (51). However, these changes likely reflect more than physical strengthening alone. This type of training is specifically designed to reduce cognitive load (52), increase confidence in prosthetic use (31), and decrease fear-based compensatory behaviours (53), all of which can positively influence gait. As such, improvements in psychological readiness and trust in the prosthetic limb may have contributed to enhance prosthetic-side gait dynamics alongside any physical adaptations.

The concurrent reduction in anterior-posterior and mediolateral centre-of-pressure displacement on the prosthetic side, together with increased prosthetic step length, likely reflects a smoother, less compensatory stance phase of the prosthetic limb. In particular, the reduction in mediolateral centre of pressure displacement suggests improved pelvic stability during prosthetic-side loading (54). These prosthetic-side improvements, faster walking speed, increased step length, and greater range of motion combined with lower centre-of-pressure excursions, indicate a more stable prosthetic-side gait pattern with fewer compensatory movements (17). Although sound-side gait parameters were not collected, the observed prosthetic-side changes align with established strategies for improving balance and mobility in transtibial amputees (25, 35, 47, 55–59), a population that typically experiences declining physical capability while simultaneously facing increased mechanical work demand during daily tasks (16).

While the changes in performance, range of motion, and centre-of-pressure occurred in both groups the magnitude of improvement differed substantially. The dual-task group demonstrated greater gains in dual-task walking performance time, although this difference did not reach statistical significance (Figure 2A). Significant *post-hoc* effects were present for prosthetic limb step length, joint range of motion and centre-of-pressure displacement in both the mediolateral and anterior-posterior directions. Taken together, these prosthetic-side improvements indicate smoother and a more stable gait pattern with fewer compensatory movements, consistent with the added benefit of cognitive-motor engagement in the dual-task intervention group (38).

Although it may seem intuitive that the physical demands of training alone would improve a presumably automatic motor activity such as walking in a healthy population (60), key executive control processes such as attention, planning, working memory, perceptual-motor integration play a central role in regulating gait (38, 61). Particularly in individuals with transtibial amputation where sensorimotor deficits increase cognitive demands (25–28). Therefore, the use of dual-task training to pair a motor or balance task with simultaneous cognitive tasks is important for real-world higher level functioning (62).

The additional improvements observed in the dual-task group of this study support the literature where gait automaticity is reduced in transtibial amputees, necessitating increased conscious control and proximal-driven gait strategies that increase the cognitive regulation required for limb placement (27, 28, 31, 32, 35). Although gait automaticity was not monitored directly in this study, the outcome measures presented including improved performance, increased step length, greater joint range of motion, and more stable centre-of-pressure transitions are consistent with enhanced cognitive-motor engagement.

Importantly, improvements in physical function and mobility for individuals with transtibial amputation are well documented (7, 63, 64), with prosthetic device characteristics influencing functional outcomes (65). Home-based exercise programmes have also demonstrated positive post-operative effects (66), and eight weeks of corrective and postural exercise rehabilitation has been shown to improve quality of life (67). However, the data presented in Figure 5 shows that neither the control group nor single-task motor-training group improved their composite quality of life scores. This is interesting, as motor-training interventions are generally affective in enhancing functional capacity and perceived well-being (68). In this group of participants, single-task motor training improved functional capacity without an improved perception of well-being.

In contrast, participants undertaking dual-task training demonstrated improved composite quality of life scores, suggesting that the added cognitive load inherent to dual-task practice may better prepare individuals for the attentional, postural, and environmental challenges encountered outside the clinical setting. Thus, facilitating more transferable functional gains and greater perceived improvements in daily living. Future work needs to examine how dual-task training influences neuromuscular control, including its potential to normalise co-contraction patterns and reduce energy cost of walking. In addition, the role of dual-task training in facilitating prosthetic acclimation warrants investigation to determine how such training might be positioned within standard rehabilitation regimens. Finally, studies that track participants activity patterns over extended periods outside of the clinical setting are needed to establish how cognitive-motor training influences real-world functional independence and quality of life.

## 5 Conclusions

Single-task and dual-task training both improved dual-task walking performance relative to the control group, with positive training effects observed for prosthetic-limb step length, knee and hip joint ROM, and centre-of-pressure path length in the anterior-posterior and mediolateral planes. Across all gait variables, dual-task training produced greater improvements than single-task training. Only dual-task training resulted in significant pre- to post-intervention increases in composite quality-of-life scores. Given the greater overall impact of dual-task training, these findings support its incorporation into rehabilitation programmes to enhance gait performance and quality of life in individuals with unilateral transtibial amputations.

## Data Availability

The original contributions presented in the study are included in the article/Supplementary Material, further inquiries can be directed to the corresponding authors.
